# Treatment and outcome of the patients with rhabdomyosarcoma of the biliary tree: Experience of the Cooperative Weichteilsarkom Studiengruppe (CWS)

**DOI:** 10.1186/s12885-019-6172-5

**Published:** 2019-10-14

**Authors:** Cristian Urla, Steven W. Warmann, Monika Sparber-Sauer, Andreas Schuck, Ivo Leuschner, Thomas Klingebiel, Gunnar Blumenstock, Guido Seitz, Ewa Koscielniak, Jörg Fuchs

**Affiliations:** 1 Department of Pediatric Surgery and Pediatric Urology, University Children’s Hospital Tuebingen, Hoppe-Seyler-Strasse 3, 72076 Tübingen, Germany; 2 Klinikum Stuttgart, Zentrum für Kinder-, Jugend- und Frauenmedizin, Olgahospital, Pediatrics 5 (Pediatric Oncology, Hematology and Immunology), Kreigsbergstrasse 62, 70174 Stuttgart, Germany; 3 Klinikum Ingolstadt, Prostatakarzinom Zentrum, Krumenauerstraße 25, 85049 Ingolstadt, Germany; 4 Department of Pediatric Pathology, University Hospital Schleswig-Holstein, Arnold-Heller-Str. 3/14, 24105 Kiel, Germany; 5 Department of Pediatric Hematology and Oncology, University Hospital Frankfurt, Theodor-Stern-Kai 7, 60590 Frankfurt/Main, Germany; 6 Department of Clinical Epidemiology and Applied Biometry, University Hospital of Tuebingen, Silcherstraße 5, 72076 Tübingen, Germany; 7 Department of Pediatric Surgery, University Children’s Hospital, Baldingerstr, 35043 Marburg, Germany

**Keywords:** Rhabdomyosarcoma, Biliary tree, CWS Studiengruppe, Treatment, Outcome

## Abstract

**Background:**

Biliary rhabdomyosarcoma (RMS) is the most common biliary tumor in children. The management of affected patients contains unique challenges because of the rarity of this tumor entity and its critical location at the porta hepatis, which can make achievement of a radical resection very difficult.

**Methods:**

In a retrospective chart analysis we analysed children suffering from biliary RMS who were registered in three different CWS trials (CWS-96, CWS-2002P, and SoTiSaR registry).

**Results:**

Seventeen patients (12 female, 5 male) with a median age of 4.3 years were assessed. The median follow-up was 42.2 months (10.7–202.5). The 5-year overall (OS) and event free survival (EFS) rates were 58% (45–71) and 47% (34–50), respectively. Patients > 10 years of age and those with alveolar histology had the worst prognosis (OS 0%). Patients with botryoid histology had an excellent survival (OS 100%) compared to those with non-botryoid histology (OS 38%, 22–54, *p* = 0.047). Microscopic complete tumor resection was achieved in almost all patients who received initial tumor biopsy followed by chemotherapy and delayed surgery.

**Conclusion:**

Positive predictive factors for survival of children with biliary RMS are age ≤ 10 years and botryoid tumor histology. Primary surgery with intention of tumor resection should be avoided.

## Background

Rhabdomyosarcoma (RMS) is the most common soft tissue sarcoma of the childhood, accounting for about 5% of all pediatric malignancies [1]. Rhabdomyosarcoma of the biliary tree represents only about 0.5% of all pediatric RMS [1, 2]. Biliary RMS usually arises in the common bile duct, but it can originate from anywhere along the biliary tree [1–4].

Late recognition of the biliary tree malignancy, its critical location, and frequent extension into the liver are the main factors responsible for diminished survival expectancy in a tumor of otherwise favorable histology. In 1985 the Intergroup Rhabdomyosarcoma Study Group (IRSG) reported the first series of 10 cases of biliary RMS treated on IRS (Intergroup Rhabdomyosarcoma Study) I and II protocols between 1972 and 1984 [2]. At that time the outcome for patients was poor and only 4/10 patients survived. However, over the years, due to the implementation of multimodal treatment concepts the survival of patients with biliary RMS has improved, reaching a 5-year OS rate of 66% as reported by Spunt et al. [1].

The role of surgery in the treatment of patients with biliary RMS remains controversial. While some authors recommend aggressive surgical resection of the tumor [4, 5], others questioned the necessity of aggressive surgical excision for this type of tumors [1].

The objective of this study was to analyze the data of patients suffering from biliary RMS who were treated within two different Cooperative Weichteilsarkom Studiengruppe (CWS) trials and SoTiSaR (Soft Tissue Sarcoma) registry with regard to treatment concepts and outcome.

## Methods

### Patients

Charts of patients that were enrolled on the multicentre trials CWS-96 (01/07/1995–31/12/2000), CWS-2002P (01/01/2003–31/12/2007) and SoTiSaR registry (start 01/07/2009) were retrospectively analysed.

Children included in the present study met the following criteria: age 0–18 years, confirmation of biliary RMS diagnosis by central pathological review, no previous treatment for sarcoma.

All patients were treated according to the corresponding study protocols combining multi-agent chemotherapy, surgery, and/or radiation therapy (RT).

Written informed consent to participate in the study was obtained from the patients, guardians or parents by the treating physician prior to inclusion into the trial with respect to the requirements of the declaration of Helsinki and in accordance with the regulations of the respective ethics committee (LAEK No. 105/95, University Tübingen No. 51/2003, University Tübingen No. 158/2009B02). Data were retrospectively analysed with regard to patients’ characteristics, treatment modalities, and outcome.

### Definition of terms

#### Tumor localization

Rhabdomyosarcoma of the biliary tree includes tumors arising from the intra- or extrahepatic biliary ducts, gallbladder, cystic duct, and ampulla Vateri. RMS arising elsewhere in the gastrointestinal tract, in the retroperitoneum, and undifferentiated sarcoma of the liver were excluded from this analysis.

#### Tumor response to chemotherapy

Tumor volumes and lymph node involvement were assessed on initial imaging (CT scan or MRI). Response was assessed after three to four courses of chemotherapy. Complete response (CR) was defined as lack of residual tumor on post-chemotherapy radiological assessment. If there was an unclear residual structure, patients were classified as CR if no viable tumor was found upon second-look surgery or if the structure remained unchanged for at least 6 months. Good response (GR) was defined as a tumor volume regression of two thirds, poor response (PR) as a regression of one third but less than two thirds, and objective response (OR) as a regression of less than one third. Progressive disease (PD) was defined as no change or any increase in tumor volume.

#### Tumor resection status

Tumor resection status was classified as R_0_ (microscopically complete) if resection margins were microscopically free of tumor cells (IRS group I). R_1_ resection status (microscopically incomplete) was applied if microscopically detectable malignant cells were present on the resection margins (IRS group II). Surgical resection status was considered as being R_2_ (macroscopically incomplete) if gross residual disease was present after surgery (IRS group III). The IRS Groups only refer to primary surgery.

#### Surgical procedures

Surgical procedures were classified as follows:

Primary resection (up-front resection) was defined as tumor resection prior to administration of chemotherapy and/or radiotherapy.

Secondary or delayed resection was defined as resection of the tumor after neoadjuvant chemotherapy and/or radiotherapy.

#### Postoperative complications

Postoperative complications were classified according to the classification proposed by Dindo and Clavien [6].

### Treatment guidelines

#### Chemotherapy regimens

In the CWS-96 trial, high-risk patients were randomized to VAIA (vincristine, dactinomycin, ifosfamide, doxorubicin) or CEVAIE (carboplatin, epirubicin, vincristine, ifosfamide, dactinomycin, etoposide) [7, 8]. Patients treated within the CWS-2002P, assigned to the standard risk group, received IVA (ifosfamide, vincristine, dactinomycin) while the high-risk group of patients received VAIA (vincristine, dactinomycin, ifosfamide, adriamycin). Stage IV patients enrolled in the SoTiSaR registry received CEVAIE. The median duration of chemotherapy was 34 weeks (3–101 weeks).

#### Surgery

In the CWS studies, biliary RMS were assigned to the category “other localizations: retro- and intraperitoneal”. For intra- and retroperitoneal RMS, surgical guidelines recommended primary biopsy as first step. Primary tumor resection might be taken into consideration, but only if there was a reasonable chance to achieve R_0_ resection status.

Tumors of the bile ducts were highlighted as “no touch” regions because of the difficulties of achieving R_0_ resection status. Tumors in these regions should initially only undergo biopsy and subsequent chemotherapy with or without radiotherapy before definite resection.

#### Radiotherapy

Radiotherapy was indicated in patients who underwent incomplete primary or secondary resections (IRS group II and III) as well as in all patients with alveolar histology. It was not used in patients with embryonal histology who underwent an initial complete resection (IRS I). Radiation techniques were described in the respective protocol. Hyperfractionated accelerated radiotherapy (2 × 1.6 Gy/day) was performed during the 4th chemotherapy cycle (weeks 7–10) or after systemic therapy. The recommended radiation doses ranged from 30.6 Gy to 44.8 Gy depending on the extent of surgery and response to chemotherapy. Individual adaptations for very young patients were made after consultations with the CWS Study Centre and CWS reference radiotherapists.

### Statistical analyses

Statistical analyses were performed using SPSS software (version 23.0, IBM Corp. Armonk, New York, USA). Demographic data are reported as medians (interquartile ranges). The 5-year overall survival (OS) and event-free survival (EFS) rates were calculated using the Kaplan-Meier estimates (± 1 standard error, SE). For OS, the time from primary diagnosis to death (therapy-related or for other reasons) or the last follow-up was used. For EFS, the end-point was defined as the time from diagnosis to first event or last follow-up. For comparison of EFS levels, the log-rank test was used in univariate analysis. A *p*-value less than 0.05 was considered statistically significant. The survival curves were truncated on the right hand side at 5 years of follow-up because only a small proportion of the original sample remained in the study.

## Results

Since 1981, 17 (5 male, 12 female) out of > 3500 patients (0.5%) with soft tissue sarcoma registered in the prospective CWS trials and in the SoTiSaR registry suffered from RMS of the biliary tree and fulfilled the inclusion criteria for this analysis (Table 1). Median age at diagnosis was 4.33 years (1.76–10.54), median follow-up was 42.23 months (10.76–202.5). Two patients had an alveolar RMS (RMA), 15 patients had an embryonal RMS (RME). Of these 15 patients with embryonal histology, 6 patients had a botryoid subtype. Fifteen patients were below 10 years of age, while 2 patients were older than 10 years of age. Six patients had tumors smaller than 5 cm, whereas 11 patients had tumors larger than 5 cm. Patient’s characteristics are detailed in Table 1, an overview regarding treatment and outcome is given in Table 2.

**Table 1:** Patients characteristics and outcome according to histological subtypes. RME = embryonal Rhabdomyosarcoma; RME_botr._ = botryoid embryonal Rhabdomyosarcoma; RMA = alveolar Rhabdomyosarcoma; DOD = died of disease

	n	Tumor size > 5 cm	Age > 10y	Metastases	Relapse		DOD
					Local	Combined	
RME	9	6	0	3	4	1	4
RME_botr._	6	3	0	0	1	0	0
RMA	2	2	2	2	1	1	2

**Table 2:** Summary of the treatment and outcome

ID	Study	Primary surgery (IRS)	Secondary surgery	CT	Response	RT (Gy)	Relapse	Outcome
1	CWS-96	Resection (R2, III)	Re-resection (R1)	VAIA	PTR	30.4	LR	DOD
2	CWS-96	Resection (R2, III)	Re-resection (R1)	VAIA	PTR	44.8		NED
3	CWS-96	Open biopsy (R2, III)	Resection (R0)	CEVAIE Trofosfamid/VP16 Trofosfamid/Idarubicin SCT	SD			NED
4	CWS-96	Resection (R2, III)		VAIA/VACA	PTR	39.6		NED
5	CWS-96	ERCP biopsy (R2, III)		CEVAIE + maintenance therapy Vinblastin/Cyclofosfamide	CR			NED
6	CWS-96	Open biopsy (R2, III)		VAIA	GR	24	LR	NED
7	CWS-96	Resection (R1, II)		VAIA	PTR	36	LR	NED
8	CWS-96	Open biopsy (R2, III)	Resection (R1)	VAIA	PR	32.4		NED
9	CWS-96	Open biopsy (R2, III)		CEVAIE + SCT	CR	44	CR	DOD
10	CWS-96	Open biopsy (R2, III)		CEVAIE + OTIE	PD		PD	DOD
11	CWS-2002P	Open biopsy (R2, III)		VAIA	GR	30.6		NED
12	CWS-2002P	ERCP biopsy (R2, III)	Resection (R0)	I2VA	GR		CR	DOD
13	CWS-2002P	Open biopsy (R2, III)	Resection (R0)	VAIA	Nos		LR	DOD
14	SoTiSaR	Open biopsy (R2, III)	Resection (R0)	CEVAIE	SD			NED
15	SoTiSaR	ERCP biopsy (R2, III)	Resection (R0)	CEVAIE+OTIE	CR			NED
16	SoTiSaR	Resection (R0, I)		VA	PTR			NED
17	SoTiSaR	Open biopsy (R2, III)		CEVAIE + OTIE	PD		PD	DOD

*CEVAIE* ifosfamid, carboplatine, epirubicine, vincristine, dactinomycin, etoposide, *CR* complete response, *CT* chemotherapy, *DOD* dead of disease, *ERCP* endoscopic retrograde cholangiopacreatography, *GR* good response, *Gy* gray, *I2VA* ifosfamide, vincristine, dactinomycin, *NED* no evidence of disease, *Nos* not otherwise specified, *O-TIE* oral maintenance therapy – trofosfamide, idarubicine, etoposide, *PD* progressive disease, *PR* poor response, *PTR* primary tumor resection, *RT* radiotherapy, *SCT* stem cell transplantation, *SD* stable disease, *VA* vincristine, actinomycin, *VAIA* ifosfamide, vincristine, adriamycin, dactinomycin, *VP16* etoposide

Five patients presented with distant metastases at diagnosis (Table 1). In 2 patients the metastases disappeared after chemotherapy, in one patient (ID 10) pulmonary metastases were surgically removed at the time of surgery for the primary tumor via a two-cavity approach. In the remaining two patients a progression of the disease was encountered.

### Surgical approach

#### Primary resection (*n* = 5)

Primary tumor resection was performed in 5/17 patients, microscopic complete tumor resection (R_0_) was achieved in only one of these patients. Resection status was R_1_ in one patient and R_2_ in 3 patients. In two of the 4 patients with incomplete primary tumor resection the tumor was initially misjudged as choledochal cyst.

Lymph node sampling was carried out in 4 patients; positive lymph nodes were detected in two of the four children.

#### Secondary resection

**Fig. 1:**
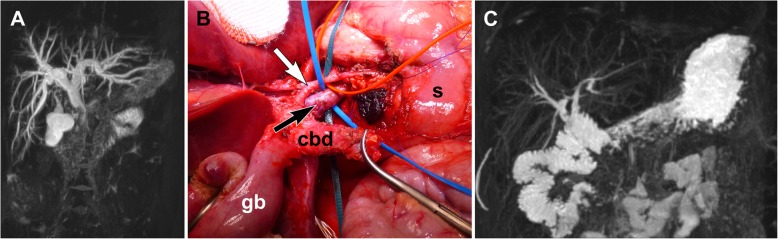
Rhabdomyosarcoma of the biliary tract. **a** Preoperative MRI image showing the tumor located at the distal region of the common bile duct. **b** Intraoperative aspect of the tumor region**. c** Postoperative follow-up MRI image showing the biliodigestive anastomosis and no evidence of local relapse**.** Abbreviations and symbols in the figure: cbd: common bile duct; gb: gallbladder; s: stomach; white arrow: hepatic artery; dark arrow: portal vein

Six patients underwent secondary tumor resection after primary biopsy and neoadjuvant chemotherapy (Fig. 1). Complete resection (R_0_) was achieved in 5/6 patients, while the remaining patient underwent an incomplete resection (R_1_). Lymph node dissection was carried out in 5 patients (N0: *n* = 4, Nx: *n* = 1).

#### Patients without tumor resection

Six patients did not undergo primary or secondary tumor resection: two patients developed tumor progression (RMA: n = 1, RME: n = 1), two patients achieved a complete response after chemotherapy (RMA: n = 1, RME: n = 1), and two patients achieved a good response after chemotherapy and received only RT. The patient with RMA who achieved complete response after chemotherapy also underwent RT.

### Postoperative complications

One patient developed a biliary peritonitis (grade II) after initial open tumor biopsy. Complications after primary resection were encountered in two patients: one exocrine pancreatic insufficiency after a subtotal resection of the pancreas (grade II), and one impaired vascularization of the segment IV of the liver after an extended left hepatectomy (grade IIIb).

Grade II postoperative complications after secondary tumor resection were noted in 3 cases: one portal vein thrombosis after a left hepatectomy, one exocrine pancreatic insufficiency after Whipple procedure, and one reactive pancreatitis after resection of the extrahepatic biliary tree.

### Radiotherapy

Radiotherapy was administered in 8/17 patients (RME: *n* = 7, RMA: *n* = 1), in 5 patients following incomplete (R_1_/R_2_) primary or secondary resection and in three patients (RME: *n* = 2, RMA: n = 1) as only measure of local treatment. The median dose of radiotherapy used was 34.2 Gy (24–44.8 Gy).

### Tumor relapses

After first complete remission confirmed by post treatment imaging, tumor relapses were observed in 6 patients, the median time to relapse was 10.5 months (6–26). Two of the 6 patients with relapses had undergone incomplete resection (R_1_ or R_2_) during the initial treatment. Another two had had complete resection (R_0_), and two had received tumor biopsy followed by solitary radiotherapy as local treatment.

Relapses were local in four patients (1 with botryoid histology, 3 with RME) of whom two initially had an incomplete resection. Relapses were combined in 2 patients (1 porta hepatis plus axillary lymph nodes, 1 periesophageal, subhepatic, peritoneal sarcomatosis, and diffuse lymph node involvement), of which one was initially treated only with chemo- and radiotherapy (RMA) and the other had a complete tumor resection (RME).

Two of the 4 patients with local relapse developed a 2nd and a 3rd relapse. Four of 6 patients with relapse died (2 with combined relapse, 2 with local relapse) and two are alive. The patients who died had alveolar (*n* = 1) and embryonal histology (*n* = 3).

### Outcome

**Fig. 2:**
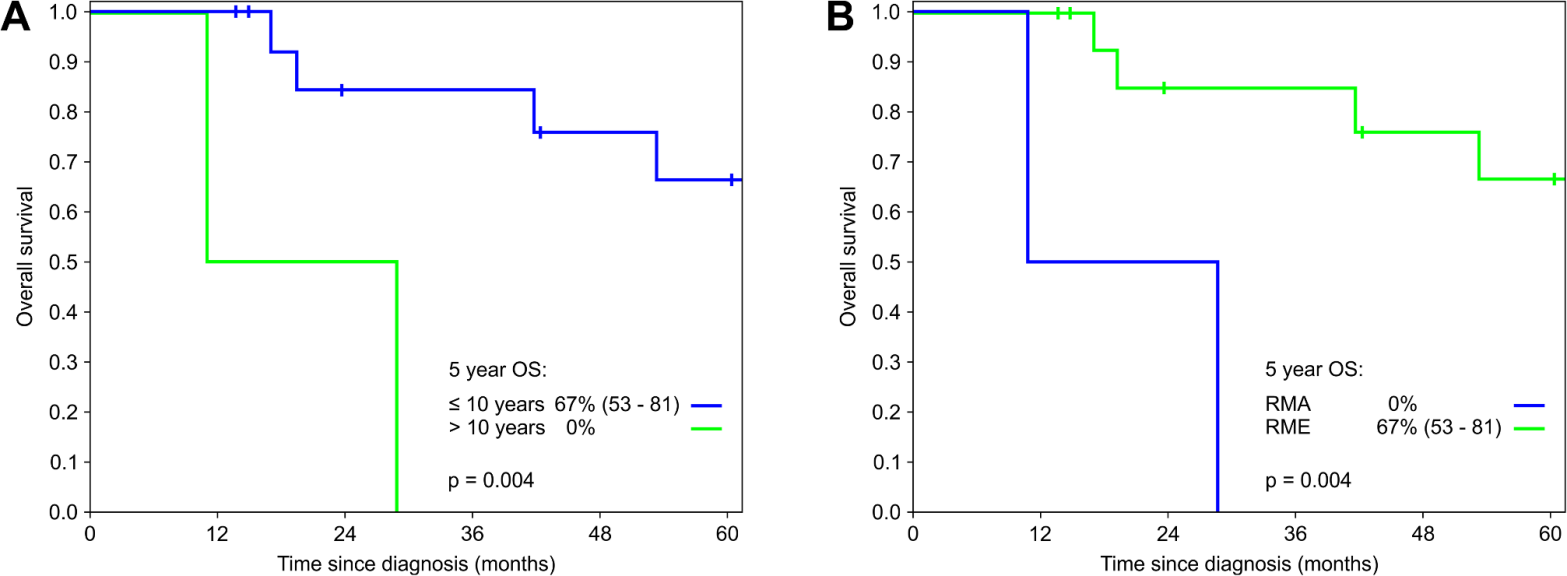
Outcome related to age (**a**) and histology (**b**)

The 5-year OS and EFS for the whole group were 58% (45–71) and 47% (34–50), respectively. Age was a prognostic predictive factor with a 5-year OS of 67% (53–81) and EFS of 54% (40–68) for patients ≤10 years of age and 0% for those > 10 years of age (*p* = 0.004 and *p* = 0.03, respectively) Fig. 2a.

Histology also was a positive predicting factor for survival. Patients with RME had significantly better survival rates (OS 67%, 53–81; EFS 54%, 40–68) compared to those with RMA (OS and EFS 0%, *p* = 0.004 and *p* = 0.03, respectively) Fig. 2b. Patients with botryoid histology had the best overall survival (100%) compared to those with non-botryoid histology (RMA + RME non-botryoid, OS 38% (22–54), *p* = 0.047). After exclusion of the patients with alveolar histology from the analysis, the patients with botryoid RME still had better survival rates compared to those with non-botryoid RME (OS: 100% **vs** 47%, 28–66, *p* = 0.084; EFS: 83%, 68–98 **vs** 38%, 21–55, *p* = 0.25).

Although there was a trend towards better outcome in patients with smaller tumors, tumor size was not a statistically significant predictive factor for survival. Overall survival and EFS in children with tumors below 5 cm were 75% (54–96) and 67% (40–94), respectively, compared with OS rates of 52% (36–68, *p* = 0.351) and EFS rates of 34% (19–49, *p* = 0.11) in children with tumors larger than 5 cm.

For the whole cohort, there was no difference in survival between patients who received RT (EFS 47%, 28–66) and those who did not receive RT (EFS 49%, 30–68, *p* = 0.84).

There was no statistically significant difference of survival rates for patients who underwent primary vs delayed tumor resection (OS 75%, 53–97 vs 63%, 42–84, *p* = 0.75; EFS 50%, 25–75% vs 67%, 48–86, *p* = 0.720). However, all patients except one undergoing primary resection received radiotherapy, whereas only one child of those undergoing delayed surgery was irradiated.

Lymph node status at diagnosis did not have an influence on survival (N0: OS 74%, 58–90; EFS 54%, 36–72; N1: OS 25%, 3–47; EFS 25%, 3–47; *p* = 0.054 and *p* = 0.189, respectively).

Patients with localized disease (IRS I-III, *n* = 12) had better survival rates (OS 63%, 42–84; EFS 37%, 17–57) compared to patients with metastatic disease (IRS IV, *n* = 5; OS 40%, 18–62; EFS 40%, 18–62), however this was not statistically significant (*p* = 0.12 and *p* = 0.49, respectively).

## Discussion

Overall, the prognosis of children with RMS has been markedly improved during recent years. One of the major contributions to this development has been achieved through the implementation of multimodal treatment concepts based on more and more specific risk stratifications [9, 10]. Tumor localization is a well-known factor that influences the outcome of children suffering from this malignancy. OS and EFS in our series of biliary RMS were only 58 and 47%, respectively, which is in accordance to the previously reported 5-year OS rate of 66% in the IRS I-IV protocols. The slightly different outcome rates are in our view caused by the less detailed histological differentiation. Explanations for the relatively low survival rates of a tumor with otherwise favorable histology are rarity of the disease, late recognition, and the critical location at the porta hepatis with extension into the liver [9–11].

Several observations from our study represent important new aspects with regard of this RMS subtype.

All patients above 10 years of age and patients with alveolar subtype of biliary RMS had the worst prognosis. None of the children in these two groups survived the disease. Although the alveolar subtype seems very rare in the biliary localization, new treatment approaches seem urgently necessary. The same holds true for children above 10 years of age. On the other hand, overall survival was 100% in patients with the botryoid subtype of biliary RMS. The selective analysis of botryoid biliary RMS has not been reported by now. Only one patient with this histological subtype had a local relapse in our study. In this regard it is furthermore remarkable that there was no statistically significant difference between outcomes of botryoid and embryonal RMS with regard to local treatment.

A problematic course has been observed in children undergoing primary resection. A high rate of incomplete resections was present in this cohort leading to a relevant amount of tumor progression. Survival in affected patients was achieved mainly through intensified treatment regimens, especially local irradiation. The vast majority of surviving patients within the IRS trials (1972–1988) who had macroscopic residual disease after surgery underwent additional local radiotherapy (mean dose 38Gy). Administration of radiotherapy has to be critically evaluated in young children. Biliary RMS is often diagnosed in the age group most susceptible to late effects; median age of patients in our study was just over 4 years. Experiences from other pediatric malignancies contain important aspects in this regard. For example, the National Wilms’ Tumor Study Group (NWTS) observed that following application of radiotherapy (> 15 Gy) to the liver there was a strong association with the development of portal hypertension in children with nephroblastoma [12]. Mulder et al. (2013) reported that liver irradiation was significantly associated with hepatic adverse effects in a large cohort of childhood cancer survivors (*n* = 1404, follow-up 12 years) [13]. Furthermore, the British Childhood Cancer Survivor Study revealed that all Wilms Tumor survivors who developed a digestive second primary neoplasm had received abdominal radiotherapy [14]. As consequence, irradiation should be cautiously considered as local treatment approach in children with biliary RMS. In almost all patients of our study, R_0_ resection status could be realized after primary tumor biopsy followed by chemotherapy and delayed resection.

Surgery has a relevant importance in the treatment of children with biliary RMS. Because of its possibly challenging character, surgery of biliary RMS in children should be executed by surgeons with a broad experience in oncological, hepatic, and biliary procedures. In some instances operating on this tumor can make complex procedures necessary- even after chemotherapy - and surgeons should be prepared to perform for example vascular and biliary reconstructions. Paganelli even reported on the case of a child who underwent Liver Transplantation for an unresectable biliary RMS [15]. Complete tumor resection with microscopically negative margins should be the main goal of surgery. As a consequence this means for example, if surgery is performed for suspicion of another reason (choledochal cysts or others) and intraoperatively a solid mass related to the biliary system is detected, then a biopsy should be taken and the decision on how to further proceed should be made with knowledge of the definite histology.

Although it still proves difficult to draw robust conclusions from a statistical point of view, it has been demonstrated in our analysis as well as in previous studies that children should undergo operations executed by experienced surgeons. Furthermore, a centralized treatment in centers of excellence should be strongly considered. Biliary RMS thus represents an important example for the necessity of homogenizing the different international trial protocols on this malignancy. Recent developments undertaken by major trial groups (COG, EpSSG, CWS) already show positive results in this regard.

## Conclusions

Taking our observations of treatment approaches and histological assessments together, we recommend the following approach in children with biliary RMS: Tumor biopsy should be the first step, either carried out surgically or via ERCP. The latter approach should be considered if obstructive jaundice is present since it also allows insertion of a stent to the biliary duct. Biopsy should then be followed by chemotherapy. As third step, local treatment should be applied. Surgery without RT should be performed in resectable botryoid RMS as well as in completely resected RME, whereas surgery plus irradiation should be administered only in incompletely resected RME and in RMA (independent of resection status). If tumors are unresectable after chemotherapy, radiotherapy should be performed as first step of the local treatment.

## Data Availability

The datasets used and/or analysed during the current study are available from the corresponding author on reasonable request.

## Abbreviations

CEVAIE: Chemotherapy regimen consisting of carboplatin, epirubicin, vincristine, ifosfamide, dactinomycin, etoposide
COG: Children’s Oncology Group
CR: Complete Response
CWS: Cooperative Weichteilsarkom-Studie (Cooperative Soft Tissue Sarcoma Study)
EFS: Event-free Survival
EpSSG: European Pediatric Soft Tissue Sarcoma Group
GR: Good Response
IRS: International Rhabdomyosarcoma Study
IRSG: International Rhabdomyosarcoma Study Group
IVA: Chemotherapy regimen consisting of ifosfamide, vincristine, dactinomycin
OR: Objective Response
OS: Overall Survival
PD: Progressive disease
PR: Poor Resonse
RMA: Alveolar subtype of Rhabdomyosarcoma
RME: Embryonal subtype of Rhabdomyosarcoma
RMS: Rhabdomyosarcoma
SoTiSaR: Soft Tissue Sarcoma Registry
VAIA: Chemotherapy regimen consisting of vincristine, dactinomycin, ifosfamide, doxorubicine
